# Use of prescription stimulant for Attention Deficit Hyperactivity Disorder in Aboriginal children and adolescents: a linked data cohort study

**DOI:** 10.1186/s40360-015-0035-8

**Published:** 2015-12-09

**Authors:** Manonita Ghosh, C. D’Arcy J. Holman, David B. Preen

**Affiliations:** Centre for Health Services Research, School of Population Health, University of Western Australia, 35 Stirling Highway, Crawley, WA 6009 Australia

## Abstract

**Background:**

Increasing recognition of Attention Deficit Hyperactivity Disorder (ADHD) among Aboriginal children, adolescents and young adults is a public health challenge. We investigated the pattern of prescription stimulants for ADHD among Aboriginal individuals in Western Australia (WA).

**Methods:**

Using a whole-population-based linked data we followed a cohort of individuals born in WA from 1980–2005, and their parents were born in Australia, to identify stimulant prescription for ADHD derived from statutory WA stimulant prescription dispensing between 2003 and 2007. Parental link was ascertained through WA Family Connections Genealogical Linkage System. Cox proportional hazards regression (HR) models were performed to determine the association between stimulant use and Aboriginal and non-Aboriginal status.

**Results:**

Of the total cohort of 186,468, around 2 % (*n* = 3677) had prescription stimulants for ADHD. Individuals with both Aboriginal parents were two-thirds (HR 0.33, 95 % CI 0.26–0.42), and with only Aboriginal mother were one-third (HR 0.69, 95 % CI 0.53–0.90) less likely to have stimulants, compared to individuals with non-Aboriginal parents. HR in Aboriginals was 62 % lower (HR 0.35, 95 % CI 0.25–0.49) in metropolitan areas, and 72 % lower (HR 0.28, 95 % CI 0.20–0.38) in non-metropolitan areas, than non-Aboriginals. The risk for simulant use was four times higher among Aboriginal boys than Aboriginal girls (HR 4.08, 95 % CI, 2.92–5.69).

**Conclusion:**

Aboriginal cultural understanding of ADHD and attitude towards stimulant medication serve as a determinant of their access to health services. Any ADHD intervention and policy framework must take into account a holistic approach to Aboriginal culture, beliefs and individual experience to provide optimal care they need.

**Electronic supplementary material:**

The online version of this article (doi:10.1186/s40360-015-0035-8) contains supplementary material, which is available to authorized users.

## Background

Attention Deficit Hyperactivity Disorder (ADHD) has been defined as a common childhood-onset neurodevelopmental disorder characterized by severe inattention, impulsivity and hyperactivity which can be associated with significant educational and social impairment [1]. Psychostimulant medications such as methylphenidate and dexamphetamine are often recommended as a first-line modality for treating ADHD [2]. Despite extensive research into factors contributing to ADHD, the aetiology and pathogenesis of the condition are poorly understood. It may be influenced by a combination of genetic and environmental factors [3–5]. As is true with most mental and developmental disorders, there is not a definitive test for ADHD, because diagnosis and classification primarily rely on observed or self-reported behaviours. Moreover, the interpretations of the severity of those behaviours and whether they should be described as abnormal are subjective [6].

In Australia, there has been an increasing recognition of ADHD symptoms among Aboriginal children and adolescents than those in the non-Aboriginal population. Zubrick et al. [7] identified 15 % Aboriginal children compared to 9.7 % non-Aboriginals at the same age were at high risk of clinically significant hyperactivity. Yet, we do not have a clear understanding of the determinants that may account for this disparity. People with ADHD are over-represented in criminal justice system [8], and the rates of incarceration are reported high among Aboriginal young [9]. The prevalence of ADHD is higher among people living in low socioeconomic condition [10, 11]. It is well established that Aboriginal children are socially and economically disadvantaged with a lower life expectancy and less than equal opportunity. Whether the higher manifestation of ADHD symptoms in Aboriginal children and adolescents is a true prevalence of clinical ADHD, or their unique learning and behavioural patterns [12] that may erroneously lead to ADHD diagnosis pause a question.

There remains a dearth of research examining the degree to which ADHD behaviour is perceived as a problem and stimulant treatment is sought for ADHD in Aboriginal communities. Aboriginals place a holistic concept of mental illness including culture and spirituality, family and community kinships, historical, social and economic factors, fear, education and loss [13] which may construct a different attitude towards Western biomedical diagnostic labels and treatment for ADHD behaviour to that of mainstream Australians. This study reports the first whole-population-based Australian study of prescription stimulant pattern for ADHD among Aboriginals. In this paper the term “Aboriginal” encompasses both Aboriginal and Torres Strait Islanders as was approved as appropriate to use in scientific publications [14].

## Methods

### Study population

**Fig. 1:**
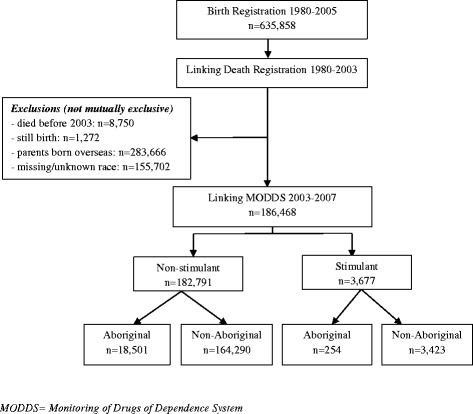
Aboriginal and non-Aboriginal cohort identification, group selection and exclusion criteria

The study population comprised a retrospective cohort of all children, adolescents and young adults who were born in WA from 1980–2005, and their parents were born in Australia, and were stratified by their parents’ Aboriginal and non-Aboriginal status. The cohort was then followed through to identify their first commencement of prescription stimulant for ADHD between 2003 and 2007. Records for still-births, parents born overseas, unknown/missing Aboriginal identity and death before 2003 were excluded, leaving 186,468 individuals for analysis. The selection criteria and process are shown in Fig. 1.

### Data sources

Data were extracted from the WA Register of Birth, Death Registry, Midwives’ Notification System (MNS) and Monitoring of Drugs of Dependence Systems through WA Data Linkage System which links databases using probabilistically matching techniques [15], and is known to achieve high level of linkage sensitivity (95–99 %) and specificity (98–99 %) [16]. The WA Family Connections Genealogical Database was linked to ascertain parent–child relationships [17].

### Variables and measurements

The outcome measure was at least one record of prescription stimulant (methylphenidate and/or dexamphetamine) dispensing for ADHD at any time during 2003–2007. Data was collected on a range of demographic factors including sex, Aboriginality, geographical remoteness, socioeconomic disadvantage and mother’s age. Parents’ Aboriginal status based on self-identification was derived from birth registry and MNS. The birth registry collects Aboriginal status of both parents, while the MNS collects information of the mothers only. Parents were recorded in birth registry as ‘Aboriginal’ ‘Aboriginal/TSI’, ‘Torres Strait Islander’, ‘Yes Aboriginal’, ‘Not Aboriginal’, ‘unknown’, and MNS data was coded as ‘Aboriginal/TSI’, ‘Caucasian’ and ‘other’. For this analysis all ‘Aboriginal’ and ‘Torres Strait Islander’ records were referred to as Aboriginal; and ‘Caucasian’, ‘Not Aboriginal’ and ‘other’ records as non-Aboriginal. Aboriginal people are known to be under-identified or misidentified due to the fact that people may not be prepared to disclose their Aboriginal status depending on the situation [18]. Therefore, parents was considered Aboriginal if they had at least one record showing as an Aboriginal/or Torres Strait Islander in either birth registry or MNS datasets. As such, a parent, identified as Aboriginal in one dataset and non-Aboriginal in the other, was considered as Aboriginal to maximise reporting of Aboriginal people in this study [19].

Geographical remoteness was measured using the Accessibility/Remoteness Index of Australia (ARIA) [20] of the Australian Census, using whichever of the 1996, 2001 or 2006 indices were closest to the year of cohort entry. ARIA scores were grouped into three levels: metropolitan, rural and remote, with metropolitan used as the reference category indicating high accessibility according to residential postcode at the time of birth. Socioeconomic disadvantage was ascertained according to the Index of Relative Socio-Economic Disadvantage (IRSD), a summary measure of Socio-Economic Indexes for Areas (SEIFA) that focused on disadvantage in terms of accessibility to education, employment and income [21]. The IRSD scores were then groups into quintiles ranging from most disadvantaged to least disadvantaged. Similar to ARIA, SEIFA score was derived from the national census years 1996, 2001, or 2006, using the index closest to the time of birth.

### Statistical analysis

Descriptive statistics were calculated for all baseline demographic characteristics of the study sample, stratified by stimulant use group. The associations between stimulant use and potential predictors including gender, age, Aboriginality, geographical remoteness and socioeconomic disadvantage were investigated using univariate and multivariate Cox proportional hazards regression (HR) models with a follow-up time 31 of December 2007. Multiple linear regression models were also fitted to compare ages of individuals at initial stimulant use during 2003–2007. A two-sided *p*-value of <0.05 was considered statistically significant in all analyses. Missing values for each variable were entered as a separate exposure category in order to include all subjects in the analyses. Statistical analyses were performed with SPSS statistical software version 21.0.

### Ethics approval

The study protocol adhered to guidelines for ethical conduct of Aboriginal health research, and was approved by the WA Aboriginal Health Ethics Committee (Ref.no. 589), Human Research Ethics Committee of University of WA (Ref.no. RA/4/1/2000), and Department of Health WA Human Research Ethics Committee (Ref.no. 2008/25). As de-identified data was utilised in this study, individual consent was not required.

## Results

Of the total cohort of 186,468 approximately 2 % (*n* = 3677) of individuals had records of prescription stimulant for ADHD during the study period. Table 1 shows the socio-demographic profile of the stimulant and non-stimulant groups. The age at initial stimulant use ranged from 2–25 years with a mean age 8.7 years (SD 2.3). Individuals who had stimulant were mostly male (*n* = 2946, 80.1 %), with non-Aboriginal parents (*n* = 3423, 93.1 %), living in metropolitan areas (*n* = 2212, 60.2 %), and were least disadvantaged (*n* = 1299, 35.3 %). Some 155 (19.5 %) individuals had at least one Aboriginal parent, represented by only an Aboriginal father in 93 instances (2.5 %), only an Aboriginal mother in 62 (1.7 %) and by both parents being Aboriginal in 99 (2.7 %).

**Table 1:** Baseline demographic characteristics of Aboriginal and non-Aboriginal children and adolescents born in WA between 1980–2005

Characteristics		No stimulant used (%)	Stimulant used for ADHD (%)
Participants		182791	3677
Parents Aboriginal status	Non-Aboriginal parents	164290 (89.9)	3423 (93.1)
	Both parents Aboriginal	10737 (5.9)	99 (2.7)
	Only father Aboriginal	3955 (2.2)	93 (2.5)
	Only mother Aboriginal	3809 (2.1)	62 (1.7)
Mothers’ age at birth	<20	11642 (6.4)	345 (9.4)
	20–24	35823 (19.6)	992 (27.0)
	25–29	60471 (33.1)	1157 (31.5)
	30–34	53085 (29.0)	834 (22.7)
	35–39	18764 (10.3)	299 (8.1)
	≥40	2762 (1.5)	44 (1.2 %)
	Unknown	244 (0.1 %)	6 (0.2 %)
Sex	Male	92708 (50.7)	2946 (80.1)
	Female	90081 (49.3)	731 (19.9)
	Unknown	2 (0.01)	0
Geographical remoteness	Metropolitan	105567 (57.8)	2212 (60.2)
	Rural	40332 (22.1)	799 (21.7)
	Remote	14160 (7.7)	171 (4.7)
	Unknown	22732 (12.4)	495 (13.5)
Socioeconomic disadvantage	Least disadvantaged	78304 (42.8)	1299 (35.3)
	Less disadvantaged	42061 (23.0)	845 (23.0)
	Little disadvantaged	17015 (9.3)	395 (10.7)
	More disadvantaged	8373 (4.6)	183 (5.0)
	Most disadvantaged	15467 (8.5)	489 (13.3)
	Unknown	21571 (11.8)	466 (12.7)

### Ethnic and demographic differences in stimulant use

Results of Cox regression analysis evaluating the associations between prescription stimulant use for ADHD and Aboriginality and other demographic characteristics are shown in Table 2. Both univariate and multivariate models showed that individuals with both Aboriginal parents were two-thirds less likely (adjusted HR 0.33, 95 % CI 0.26–0.42, *p* < 0.001) and individuals with Aboriginal mothers only were one-third less likely (adjusted HR 0.69, 95 % CI 0.53–0.90, *p* = 0.006) to use stimulants than individuals of non-Aboriginals parents. The risk for stimulant use in individuals of Aboriginal fathers was not significantly different from individuals of non-Aboriginal parents in either the crude or adjusted analysis. After adjusted, the association between risk for stimulant use and maternal age was marked. Individuals of mothers younger than aged 20 years had a 1.5 fold (HR 1.52, 95 % CI 1.33–1.74, *p* < 0.001) increased risk for stimulant use as compared to individuals of mothers aged 25–29 years, whereas a decreased risk was seen of mothers aged 30–34 years (HR 0.88, 95 % CI0.80–0.96, *p* = 0.007). Boys were nearly four times more likely to be prescribed than girls (HR 3.85, 95 % CI 3.53–4.20, *p* < 0.001). Likewise, geographical remoteness was a strong determinant of the outcomes with HR ranging from 0.87 (95 % CI 0.80–0.94, *p* < 0.001) in rural to HR 0.63 (95 % CI 0.54–0.74, *p* < 0.001) in remote areas compared with HR in metropolitan areas. Individuals with most-disadvantage had a two-fold increased risk for stimulants use compared to those with least socioeconomic status (HR 2.03, 95 % CI 1.82–2.27, *p* < 0.001).

**Table 2:** Hazard ratios and 95 % CI of prescription stimulant medication in Aboriginal and non-Aboriginal children and adolescents

Parameter		Univariate Analysis		Multivariate Analysis^a^	
		HR (95 % CI)	*P*-Value	HR (95 % CI)	*P*-Value
Parents Aboriginal status	Non-Aboriginal parents	1.0		1.0	
	Both parents Aboriginal	0.45(0.37–0.55)	<0.001	0.33(0.26–0.42)	<0.001
	Only father Aboriginal	1.13(0.92–1.38)	0.26	0.92(0.74–1.14)	0.45
	Only mother Aboriginal	0.78(0.61–1.01)	0.059	0.69(0.53–0.90)	0.006
Mothers’ age group in years at birth	<20	1.54(1.36–1.73)	<0.001	1.52(1.33–1.74)	<0.001
	20–24	1.44(1.32–1.56)	<0.001	1.42(1.30–1.56)	<0.001
	25–29	1.0		1.0	
	30–34	0.82(0.75–0.90)	<0.001	0.88(0.80–0.96)	0.007
	35–39	0.84(0.74–0.95)	0.006	0.90(0.79–1.03)	0.14
	≥40	0.84(0.62–1.13)	0.24	0.83(0.60–1.16)	0.28
Sex	Female	1.0		1.0	
	Male	3.83(3.53–4.15)	<0.001	3.85(3.53–4.20)	<0.001
Geographical remoteness	Metropolitan	1.0		1.0	
	Rural	0.95(0.87–1.03)	0.18	0.87(0.80–0.94)	<0.001
	Remote	0.58(0.50–0.68)	<0.001	0.63(0.54–0.74)	<0.001
Socioeconomic disadvantage	Least disadvantaged	1.0		1.0	
	Less disadvantaged	1.21(1.12–1.32)	<0.001	1.19(1.09–1.30)	<0.001
	Little disadvantaged	1.39(1.24–1.56)	<0.001	1.32(1.18–1.49)	<0.001
	More disadvantaged	1.31(1.12–1.53)	<0.001	1.31(1.12–1.53)	<0.001
	Most disadvantaged	1.88(1.70–2.09)	<0.001	2.03(1.82–2.27)	<0.001

^a^All parameters were included in the regression model so as to adjust each result for potential confounding by all other covariates

### Comparison of stimulant use between non-Aboriginals and Aboriginals living in metropolitan and non-metropolitan areas

In the adjusted model, individuals with both Aboriginal parents were 65 % less likely (HR 0.35, 95 % CI 0.25–0.49, *p* < 0.001) in metropolitan, and 72 % less likely in rural and remote areas (HR 0.28, 95 % CI 0.20–0.38, *p* < 0.001) to have stimulants than individuals with non-Aboriginal parents (Table 3). The HR was also lower in metropolitan, (HR 0.68, 95 % CI 0.48–0.95, *p* = 0.03) and in non-metropolitan areas (HR 0.66, 95 % CI 0.44–1.0, *p* = 0.05) for those who had only Aboriginal mothers. A 1.6 fold higher risk for stimulant use was seen in individuals of mother’s younger than 20 years old (HR 1.56 95 % CI 1.24–1.97, *p* < 0.001) compared with mother age 25–29 years old. The higher risk for stimulant use among boys was observed in both metropolitan (HR 3.69, 95 % CI 3.33–4.09, *p* < 0.001) and non-metropolitan areas (HR 4.24, 95 % CI 3.61–4.99, *p* < 0.001). HR was elevated by two-fold in the most-disadvantaged group compared with their least-disadvantaged counterparts (metropolitan – HR 2.17, 95 % CI 1.89–2.49, *p* < 0.001), (non-metropolitan– HR 1.80, 95 % CI 1.49–2.17, *p* < 0.001).

**Table 3:** Comparison of stimulant medication in Aboriginal and non-Aboriginal children by metropolitan and non-metropolitan areas

Parameter		Multivariate Analysis		Multivariate Analysis^a^	
		Metro		Non-Metro	
		HR (95 % CI)	*P*-Value	HR (95 % CI)	*P*-Value
Parents Aboriginal status	Non-Aboriginal parents	1.0		1.0	
	Both parents Aboriginal	0.35(0.25–0.49)	<0.001	0.28(0.20–0.38)	<0.001
	Only father Aboriginal	0.96(0.73–1.26)	0.76	0.8(0.55–1.17)	0.26
	Only mother Aboriginal	0.68(0.48–0.95)	0.03	0.66(0.44–1.0)	0.051
Mothers’ age group in years at birth	<20	1.51(1.28–1.78)	<0.001	1.56(1.24–1.97)	<0.001
	20–24	1.48(1.33–1.66)	<0.001	1.30(1.10–1.53)	0.002
	25–29	1.0		1.0	
	30–34	0.89(0.79–1.00)	0.04	0.85(0.71–1.01)	0.07
	35–39	0.85(0.73–1.00)	0.57	1.06(0.82–1.35)	0.67
	≥40	0.78(0.52–1.16)	0.22	1.00(0.55–1.82)	0.99
Sex	Female	1.0		1.0	
	Male	3.69(3.33–4.09)	<0.001	4.24(3.61–4.99)	<0.001
Socioeconomic disadvantage	Least disadvantaged	1.0		1.0	
	Less disadvantaged	1.19(1.07–1.33)	<0.001	1.15(0.98–1.35)	0.10
	Little disadvantaged	1.33(1.16–1.53)	<0.001	1.28(1.04–1.58)	0.02
	More disadvantaged	1.4(1.16–1.69)	<0.001	1.13(0.85–1.49)	0.41
	Most disadvantaged	2.17(1.89–2.49)	<0.001	1.80(1.49–2.17)	<0.001

^a^All parameters were included in the regression model so as to adjust each result for potential confounding by all other covariates

### Comparison of stimulant use within Aboriginal group

The fitted univariate and multivariate models for stimulant use determinants in only those individuals who had any Aboriginal parents are shown in Table 4. Aboriginal boys were four times more likely to be prescribed than Aboriginal girls (HR 4.08, 95 % CI 2.92–5.69, *p* < 0.001). Aboriginals living in remote areas were 62 % less likely (HR 0.38, 95 % CI 0.26–0.56, *p* < 0.001) to have stimulants than their city counterparts. Mothers’ age and socioeconomic status were not significantly associated with stimulant use within this group. We also fitted a multiple linear regression model to examine the association between mean age at initial prescription stimulants and demographic and geographic variables, but no association was observed (results attached as Additional file 1).

**Table 4:** Hazard ratios and 95 % CI of prescription stimulant medication in Aboriginal children and adolescents

Parameter		Univariate Analysis		Multivariate Analysis^a^	
		HR (95 % CI)	*P*-Value	HR (95 % CI)	*P*-Value
Mothers’ age group in years at birth	<20	0.70(0.47–10.4)	0.08	0.69(0.45–1.07)	0.10
	20–24	1.10(0.80–1.50)	0.56	1.20(0.85–1.69)	0.29
	25–29	1.0		1.0	
	30–34	1.06(0.72–1.57)	0.78	1.30(0.86–1.96)	0.22
	35–39	0.98(0.54–1.77)	0.94	1.25(0.68–2.28)	0.48
	≥40	0.46(0.06–3.33)	0.44	0.60(0.08–4.33)	0.61
Sex	Female	1.0		1.0	
	Male	4.51(3.27–6.23)	<0.001	4.08(2.92–5.69)	<0.001
Geographical remoteness	Metropolitan	1.0		1.0	
	Rural	0.83(0.61–1.13)	0.23	0.82(0.60–1.12)	0.20
	Remote	0.39(0.27–0.58)	<0.001	0.38(0.26–0.56)	<0.001
Socioeconomic disadvantage	Least disadvantaged	1.0		1.0	
	Less disadvantaged	1.05(0.68–1.63)	0.82	1.15(0.74–1.78)	0.53
	Little disadvantaged	1.25(0.78–1.99)	0.35	1.29(0.81–2.05)	0.29
	More disadvantaged	1.10(0.65–1.88)	0.71	1.26(0.74–2.16)	0.39
	Most disadvantaged	1.14(0.76–1.72)	0.53	1.40(0.92–2.12)	0.12

^a^All parameters were included in the regression model so as to adjust each result for potential confounding by all other covariates

## Discussion

Despite increasing recognition of ADHD among Aboriginal children [12, 22, 23], the risk of stimulant use for ADHD was markedly lower among individuals of Aboriginal parents than individuals of non-Aboriginal parents in our study. Parents are unlikely to pursue ADHD medication if they do not perceive ADHD as a clinical problem [24]. Aboriginal parents who allow children freedom to explore their environment without restrictions to make them physically and emotionally resilient [25], may perceive hyperactivity and impulsivity as normal child behaviour.

Aboriginal children were subject to removal from their families historically through systematic policy of colonial intervention, and also to a lesser extent today through out-of-homecare programs [26, 27]. Parental separation and early attachment deprivation is a risk factor for ADHD in children [28]. Aboriginal parents may attribute hyperactivity and impulsivity to child-removal-associated trauma which has been rooted in the Aboriginal cultural memory [29]. This trauma has been advanced as a reason why treatment may appear to the Aboriginal parents as a repetition of the colonial practices [30], jeopardising abilities to fulfil their roles in family and community [31].

Stimulant use was notably lower in individuals of Aboriginal mothers than fathers, possibly due to the fact that Aboriginal women traditionally play a central role in family and community, and are solely responsible for caretaking and early child socialization [32, 33]. Conversely, another study reported fathers more than mothers were associated with lower stimulant use in non-Anglophonic Australian communities [34]. The authors argued that fathers who were less likely to perceive ADHD as a problem than mothers were the decision-maker about child health in non-English speaking communities.

We found lower risk of stimulant use among Aboriginals in non-metropolitan than in metropolitan areas likely due to geographical disparities in healthcare service access with shortages of health-related infrastructure in rural areas in Australia [35]. Positive impact of community support and sense of belonging on protecting Aboriginal people against mental illness in both metropolitan and remote Aboriginal communities in Australia are documented [36, 37]. Yet, it is difficult to measure if the influence of the community support on mental health is greater in rural than metropolitan communities. In Canada, Currie et al. [38] reported that while Aboriginal enculturation was protective against substance use and strengthened psychological wellbeing, mainstream acculturation weakens the influence of cultural ties and was a risk factor for substance abuse in urban Aboriginal adults. As Aboriginal people continue to urbanize in Australia [39], they may adopt beliefs and attitudes to ADHD medication of the mainstream urban society leading to the discrepancy in stimulant use between metropolitan and remote Aboriginal communities here.

We made a number of other salient observations in this study. The first confirmed the well-known gender variation in stimulant use. Both Aboriginal and non-Aboriginal boys had elevated risk of stimulant use possibly due to the fact that boys commonly manifest hyperactivity and impulsivity [40] which may closely entwine with heuristics and gender stereotypes influencing referral [41] and diagnose [42]. Secondly, the association between young maternal age and increased stimulant use risk is well established [43–45]. A high level of maternal depression, smoking and substance use during pregnancy has been reported as risk factors for ADHD in children [44, 45]. This association however was not marked in Aboriginal groups here, and may need further research. Thirdly, associations between socioeconomic hardship and increased stimulant use was in line with previous Australian studies [34, 40]. While a high prevalence of ADHD in marginalised children is well established [10, 46], a large proportion of Australian children living in poverty were reported as being treated without meeting the ADHD diagnosis criteria [47, 48]. Hence, disadvantaged children who are more likely to be diagnosed with ADHD represent an important public health issue. Yet our findings of no association between disadvantage and stimulant use within Aboriginal groups is novel, however different interpretations exist and further investigation is warranted. One possibility is that within the Aboriginal population, social disadvantage correlates with ADHD symptoms and with a tendency not to receive treatment. Community support and cultural bond which have been shown to buffer mental and behavioural problems for marginalised people [49] could be another explanation.

Some limitations need to be considered when interpreting our results. The datasets did not permit identification of individuals diagnosed with ADHD but not prescribed stimulants. It would have been useful to examine differences between diagnosis rates and stimulant treatment in Aboriginal children to investigate the likelihood of stimulant over- or under-prescribing. In order to correctly identify Aboriginal people we triangulated information from two data sources; yet, it is still possible that Aboriginality is under-reported or misreported. It is also possible that our results were affected by unmeasured and, as yet, unidentified confounders.

## Conclusion

Lower stimulant use for ADHD in children and adolescents of Aboriginal parents in our study suggests either Aboriginal parent perceive ADHD symptoms as normal child behaviour, have a negative attitude towards medication, or cultural competency provides a coping mechanism to make the ADHD symptoms functional. Alternatively, Aboriginal children who would stand to benefit from ADHD medication may face barriers to access. Aboriginal children should be protected from misdiagnosis and over-diagnosis; however, great care should be taken to ensure full access to appropriate services when required. A better understanding of Aboriginal perceptions of ADHD and stimulant treatment is crucial to identify vulnerabilities and develop targeted interventions and policy that account for social factors and align with Aboriginal culture to provide optimal care. We suggest two avenues for future research examining ADHD prevalence in Aboriginal children with narrowing of focus: first, for the rate of ADHD diagnosis and stimulant treatment to be investigated; and second, qualitative research to explore Aboriginal perception towards ADHD and stimulant treatment.

## Additional file

Additional file 1:
**Mean age in years at initial prescription in those receiving a stimulant medication for ADHD according to cultural and demographic factors.** (DOCX 18 kb)

## Abbreviations

ADHD: Attention Deficit Hyperactivity Disorder
ARIA: Accessibility/Remoteness Index of Australia
HR: Hazards Ratios
IRSD: Index of Relative Socio-Economic Disadvantage
MNS: Midwives’ Notification System
SEIFA: Socio-Economic Indexes for Areas
WA: Western Australia
