# Supplementary material for: Use of prescription stimulant for Attention Deficit Hyperactivity Disorder in Aboriginal children and adolescents: a linked data cohort study
Source: BMC Pharmacol Toxicol. 2015 Dec 9;16:35. doi: 10.1186/s40360-015-0035-8 (PMC4673717; doi:10.1186/s40360-015-0035-8)
Supplement: Additional file 1: — Mean age in years at initial prescription in those receiving a stimulant medication for ADHD according to cultural and demographic factors. (DOCX 18 kb) [file 40360_2015_35_MOESM1_ESM.docx]

| Mean age in years at initial prescription in those receiving a stimulant medication for ADHD according to cultural and demographic factors |
| --- |

| Parameter | | Univariate Analysis | | | Multivariate Analysis | | |
| --- | --- | --- | --- | --- | --- | --- | --- |
|  |  | Mean age | Mean difference  (95% CI) | p-value | Mean age | Mean difference  (95% CI) | p-value |
| Indigenous status | Non-Aboriginal | 8.72 | 1.0 |  | 8.69 | 1.0 |  |
|  | Both parents Aboriginal | 8.63 | -0.09 (-0.55, 0.36) | 0.69 | 8.55 | -0.14 (-0.60, 0.32) | 0.55 |
|  | Only father Aboriginal | 8.33 | -0.39 (-0.85, 0.08) | 0.11 | 8.33 | -0.35 (-0.82, 0.11) | 0.14 |
|  | Only mother Aboriginal | 8.24 | -0.48 (-1.05, 0.09) | 0.10 | 8.26 | -0.43 (-1.00, 0.14) | 0.14 |
| Sex | Male | 8.70 | 1.0 |  | 8.47 | 1.0 |  |
|  | Female | 8.69 | -0.01 (-0.19, 0.17) | 0.92 | 8.45 | -0.02 (-0.20, 0.17) | 0.86 |
| Geographical remoteness | Metropolitan | 8.68 | 1.0 |  | 8.53 |  |  |
|  | Rural | 8.60 | -0.08 (-0.26, 0.10) | 0.39 | 8.47 | -0.06 (-0.25, 0.12) | 0.50 |
|  | Remote | 8.97 | 0.29 (-0.06, 0.65) | 0.10 | 8.84 | 0.30 (-0.05, 0.66) | 0.09 |
| Socioeconomic disadvantaged | Least disadvantaged | 8.74 | 1.0 |  | 8.44 | 1.0 |  |
|  | Less disadvantaged | 8.72 | -0.03 (-0.22, 0.17) | 0.78 | 8.42 | -0.02 (-0.22, 0.18) | 0.84 |
|  | Little disadvantaged | 8.51 | -0.24 (-0.49, 0.02) | 0.07 | 8.24 | -0.20 (-0.46, 0.05) | 0.12 |
|  | More disadvantaged | 8.39 | -0.36 (-0.71, -0.01) | 0.05 | 8.12 | -0.33 (-0.68, 0.03) | 0.07 |
|  | Most disadvantaged | 8.62 | -0.13 (-0.36, 0.11) | 0.28 | 8.36 | -0.09 (-0.33, 0.15) | 0.47 |
